# Pyroptosis: A New Insight Into Eye Disease Therapy

**DOI:** 10.3389/fphar.2021.797110

**Published:** 2021-12-03

**Authors:** Yun Zhang, Yan Jiao, Xun Li, Sheng Gao, Nenghua Zhou, Jianan Duan, Meixia Zhang

**Affiliations:** ^1^ Department of Ophthalmology, West China Hospital, Sichuan University, Chengdu, China; ^2^ Research Laboratory of Macular Disease, West China Hospital, Sichuan University, Chengdu, China; ^3^ State Key Laboratory of Biotherapy and Cancer Center, West China Hospital, Sichuan University, Chengdu, China; ^4^ Key Laboratory of Drug Targeting and Drug Delivery System of Ministry of Education, West China School of Pharmacy, Sichuan University, Chengdu, China

**Keywords:** pyroptosis, eye disease, inflammasome, NLRP3, pyroptosis inhibitors

## Abstract

Pyroptosis is a lytic form of programmed cell death mediated by gasdermins (GSDMs) with pore-forming activity in response to certain exogenous and endogenous stimuli. The inflammasomes are intracellular multiprotein complexes consisting of pattern recognition receptors, an adaptor protein ASC (apoptosis speck-like protein), and caspase-1 and cause autocatalytic activation of caspase-1, which cleaves gasdermin D (GSDMD), inducing pyroptosis accompanied by cytokine release. In recent years, the pathogenic roles of inflammasomes and pyroptosis in multiple eye diseases, including keratitis, dry eyes, cataracts, glaucoma, uveitis, age-related macular degeneration, and diabetic retinopathy, have been continuously confirmed. Inhibiting inflammasome activation and abnormal pyroptosis in eyes generally attenuates inflammation and benefits prognosis. Therefore, insight into the pathogenesis underlying pyroptosis and inflammasome development in various types of eye diseases may provide new therapeutic strategies for ocular disorders. Inhibitors of pyroptosis, such as NLRP3, caspase-1, and GSDMD inhibitors, have been proven to be effective in many eye diseases. The purpose of this article is to illuminate the mechanism underlying inflammasome activation and pyroptosis and emphasize its crucial role in various ocular disorders. In addition, we review the application of pyroptosis modulators in eye diseases.

## Introduction

Pyroptosis, a programmed cell death dependent on the pore-forming activity of the gasdermin protein family with an inflammatory response, plays an essential part of the body’s intrinsic immune response in antagonizing pathogen infection and sensing endogenous risk signals (Man et al., 2017; Shi et al., 2017). Cells are stimulated to form a multiprotein complex called the inflammasome that can convert inactive pro-caspase-1 to active caspase-1, which can cleave gasdermin D (GSDMD) at its central linker domain and release the N-terminal GSDMD domain, causing N-terminal GSDMD domain fragments to perforate the plasma membrane and form membrane pores, further leading to cell swelling and lytic cell death. Meanwhile, active caspase-1 processes inflammatory factors (IL-1β, IL-18, etc.) to the mature form and releases them to the extracellular matrix through ruptured membranes, conferring the proinflammatory nature of pyroptosis. Mature IL-1β acts as a potent proinflammatory mediator to recruit innate immune cells to sites of infection and regulate adaptive immune cells. Mature IL-18 can promote the secretion of interferon (IFN-γ) and enhance the cytolytic activity of natural killer cells and T cells, contributing to the clearance of pathogenic microbial infections or aberrant cells *in vivo* (Dinarello, 2009). Apoptosis, the first programmed cell death to be described, can be initiated through extrinsic and intrinsic pathways and characterized by cell contraction, nuclear condensation and division, and dynamic membrane blistering and loss. It activates the execution phase of cell death and allows the clearance of apoptotic cells without eliciting an inflammatory response, which is critical to physiological homeostasis in almost every organ system (Galluzzi et al., 2018). Indeed, a growing amount of evidence demonstrates that abnormal pyroptosis is closely related to autoimmune diseases, metabolic diseases, infectious diseases, cardiovascular diseases, neurological-related diseases, and ocular diseases (Man et al., 2017; Van Gorp et al., 2019; Zeng et al., 2019; McKenzie et al., 2020; Sharma and Kanneganti, 2021). The goal of this review is to discuss the role of pyroptosis and inflammasomes in the pathogenesis of ocular disorders and the application of pyroptosis inhibitors in eye diseases.

## The Classification of Inflammasomes

The inflammasome is defined as a complex formed by pattern recognition receptors (PRRs), apoptosis speck-like protein (ASC), and pro-caspase-1 protein when cells are stimulated by a danger signal, functioning as cleaving pro-caspase-1 to active caspase-1. In most cases, it is a critical process of pyroptosis.

**FIGURE 1:**
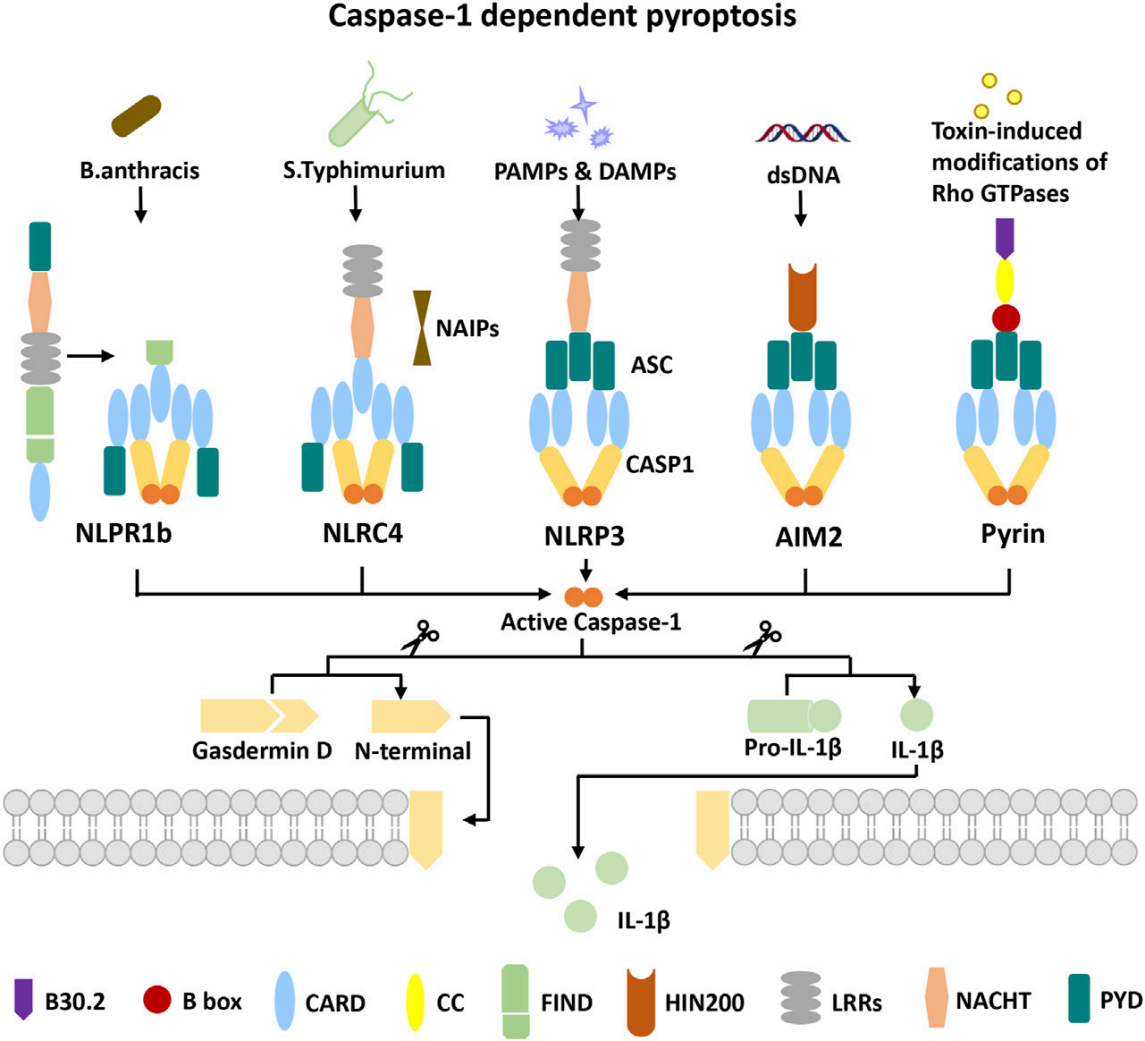
Overview of five inflammasome complexes. NLRP1 undergoes autocatalytic processing in its FIIND followed by proteasomal degradation of its autoinhibitory N-terminus to engage caspase-1 in the C-terminal CARD for its activation. NAIPs are necessary for NLRC4 inflammasome activation to recognize *S. typhimurium*. In NLRP1 and NLRC4 activation events, caspase-1 can be directly recruited independently of the adapter ASC. The NLRP3 inflammasome can be activated by a broad spectrum of exogenous and endogenous stimuli. AIM2 activation requires dsDNA of microbial or host origin in the cytosol. Toxin-induced modifications of Rho GTPases reduce the phosphorylation of pyrin, which promotes the assembly of the pyrin inflammasome. NLRP3, AIM2, and pyrin activation events all require the adaptor ASC to activate caspase-1. Finally, activated caspase-1 drives gasdermin D cleavage to release the N-terminus, which forms the gasdermin D pore and drives pyroptosis. At the same time, activated caspase-1 cleaves pro-IL-1β to mature IL-1β into the extracellular matrix.

Pattern recognition receptors (PRRs), a class of immune receptors mainly expressed in immune cells capable of identifying multiple pathogen-associated molecular patterns (PAMPs) or damage-associated molecular patterns (DAMPs) of invading microorganisms, are the first link of the innate immune system against infection. According to the homology of the protein domain, most PRRs can be classified into five families: Toll-like receptor (TLR), C-type (carbohydrate-binding lectin domain) lectin receptor (CLR), NOD-like receptor (NLR), retinoic acid–inducible gene (RIG)-I–like receptor (RLR), and AIM2-like receptor (ALR). According to protein localization, PRRs can be separated into unbound intracellular receptors and membrane-bound receptors. The former class includes NLRs, RLRs, and ALRs, which recognize the presence of intracellular pathogens in the cytoplasm. TLRs and CLRs are located on cell members or endocytic compartments, which recognize the presence of microbial ligands in the extracellular space or endosomes (Dostert et al., 2008; Brubaker et al., 2015; Plato et al., 2015). Multiple proteins of the NLR family and ALRs families have been found to form inflammasomes that mediate the occurrence of pyroptosis (Figure 1). PRRs have been found to form inflammasomes, including NLRP1, NLRP3, NLRC4, NLRP6, NLRP9, NLRP12, pyrin, AIM2, IFI16, and CARD8. NLRP3 is the most widely studied and the best characterized inflammasome.

### NLRP3

NLRP3 is composed of three domains, the PYD and NACHT and LRR domains. It can recruit and activate caspase-1 indirectly with the involvement of the adaptor protein ASC. NLRP3 can be activated by a wide variety of factors, including not only pathogen-related molecular patterns from invading pathogens such as bacterial RNA and toxin ion channel proteins but also damage-related molecular patterns such as ATP and oxidative mitochondrial DNA and multiple molecules closely associated with diseases such as β-amyloid in Alzheimer’s disease and cholesterol crystallization in atherosclerosis (Swanson et al., 2019). Therefore, the NLRP3 inflammasome plays a very important role in various human diseases, such as pathogen infection, autoimmune diseases, neurodegenerative diseases, and cancer.

**FIGURE 2:**
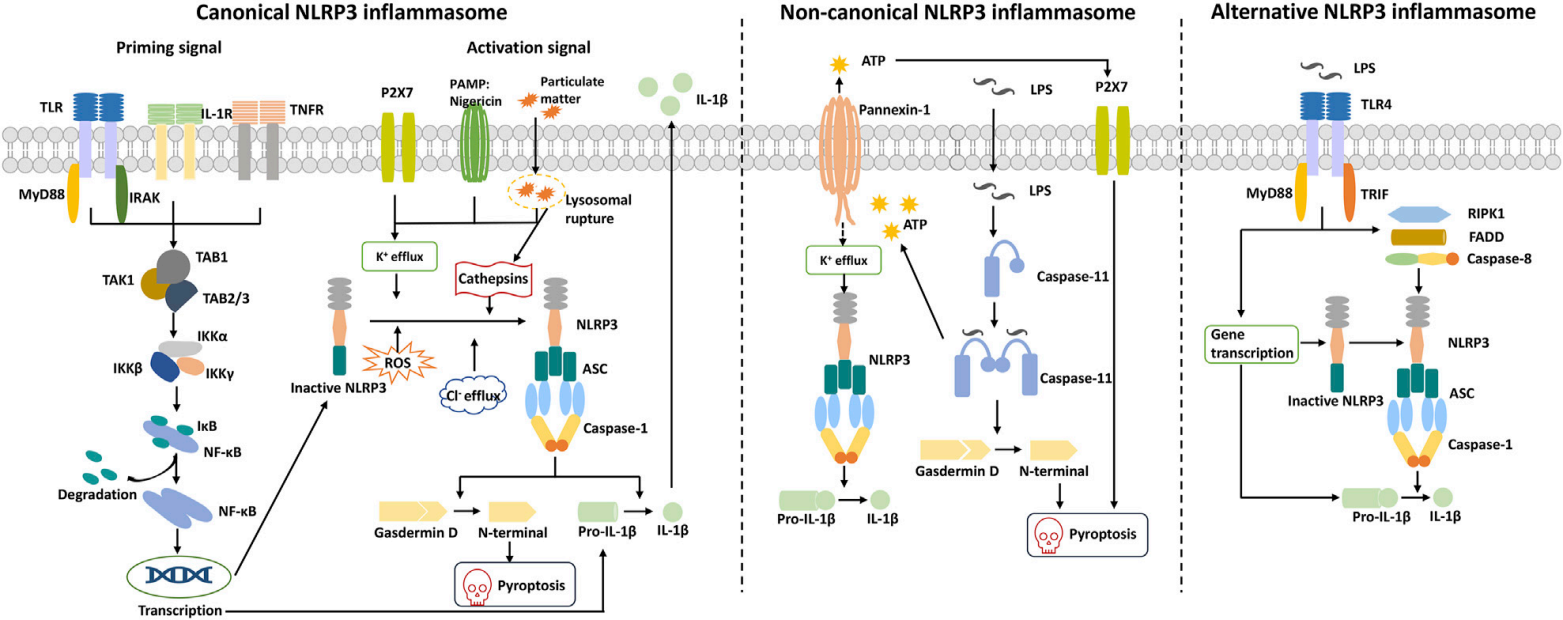
Mechanisms of NLRP3 inflammasome activation. Canonical NLRP3 inflammasome activation requires two signals: a priming signal and an activation signal. The priming signal is triggered by endogenous cytokines or microbial molecules and causes the upregulation of NLRP3 and pro-IL-1β through the activation of the transcription factor NF-κB signaling pathway. The activation signal is triggered by various stimuli, such as pore-forming toxins, particular matter, and viral RNA, which can induce K^+^ efflux, Cl^−^ efflux, mitochondrial dysfunction (ROS release), and lysosomal disruption (cathepsins). These events promote NLRP3 oligomerization to recruit ASC and pro-caspase-1 and form the activated NLRP3 inflammasome. Activated caspase-1 cleaves gasdermin D to release the N-terminal domain and induce pyroptosis. Meanwhile, caspase-1 cleaves pro-IL-1β to IL-1β and is released to the extracellular matrix. Non-canonical NLRP3 inflammasome activation is induced by cytosolic LPS released from gram-negative bacteria. LPS is delivered into the cytosol and activates caspase-11, whose activated state can trigger the pannexin-1 channel to induce K^+^ efflux and ATP release. Subsequently, the NLRP3 inflammasome is activated, and mature IL-1β is released. Activated caspase-11 also induces pyroptosis via cleavage of gasdermin D and formation of pores on the cell membrane. Alternative NLRP3 inflammasome activation occurs when human monocytes are exposed to LPS, which requires RIPK1, FADD, and caspase 8 to activate the NLRP3 inflammasome rather than K^+^ efflux. ASC speck formation and pyroptosis are also absent in this pathway.

Canonical NLRP3 inflammasome activation generally requires two signals: a priming signal and an activation signal (Figure 2). The priming signals are provided by microbial components such as lipopolysaccharide (LPS), which can be recognized by the Toll-like receptor TLR4 and can also be endogenous molecules, including TNF-α or IL-1β (Bauernfeind et al., 2009; Franchi et al., 2009). The priming signals induce NLRP3 and pro-IL-1β expressions through activation of the NF-κB signaling pathway (Gritsenko et al., 2020). The activation signal is triggered by ATP, Nigericin, silica, particulate matter, viral RNA, etc. These activation factors can induce cellular stress and activate the NLRP3 inflammasome, which is indirectly sensed by NLRP3 instead of being directly recognized by NLRP3.

Non-canonical inflammasome pathway activation is mediated through humanized caspase-4/5 or murine caspase-11, which directly identifies LPS and can be activated (Figure 2). This effect is not dependent on the conventional LPS extracellular receptor TLR4 and does not require the involvement of other receptor proteins, such as NLRP3 and the adaptor protein ASC (Kayagaki et al., 2011; Hagar et al., 2013; Kayagaki et al., 2013; Shi et al., 2014). Activated caspase-4/5/11 cleaves the pyroptosis effector protein GSDMD, which triggers pyroptosis. Then, it causes pannexin-1–mediated ATP release, triggers K^+^ efflux, and further activates the NLRP3 inflammasome (Baker et al., 2015; Kayagaki et al., 2015; Rühl and Broz, 2015; Yang et al., 2015).

Unlike macrophages, human monocytes can activate caspase-1 and secrete mature IL-1β without activation signals after LPS stimulus (Netea et al., 2009). This pathway is defined as the alternative NLRP3 inflammasome pathway independent of K^+^ efflux, and there is no evidence for ASC speck formation or pyroptosis (Gaidt et al., 2016). Studies have shown that TLR4-TRIF-RIPK1/FADD/caspase-8 signaling is involved in NLRP3-mediated inflammatory factor release and that activated caspase-8 can cleave GSDMD to lead to pyroptosis (He et al., 2013; Orning et al., 2018) (Figure 2).

To date, there is no consensus model of NLRP3 activation, and multifarious upstream signals are involved in the regulation of NLRP3 inflammasome activation, including potassium ion (K^+^) efflux, chloride ion (Cl^−^) efflux, flux of calcium ions (Ca^2+^), lysosomal disruption, mitochondrial dysfunction, metabolic changes, and trans-Golgi disassembly. These upstream signals might act in tandem or independently on NLRP3 inflammasome activation. With few exceptions, low intracellular concentrations of K^+^ are a necessary upstream event in NLRP3 activation (Muñoz-Planillo et al., 2013). Nigericin, as a perforated toxin, can directly cause K^+^ efflux. After ATP stimulation, P2X7 family receptors (non-selective cation channels) promote Ca^+^ and Na^+^ influx and coordinate with the K+ channel TWIK2 to mediate K^+^ efflux, which enhances NLRP3 inflammasome activation (Di et al., 2018). The lower intracellular concentration of Cl^−^ is also an essential signal for NLRP3 inflammasome activation by several stimuli (Nigericin, ATP, etc.), and the chloride intracellular channel (CLIC) is involved in the process (Tang et al., 2017). Nigericin-induced mitochondrial damage and ROS production can promote the plasma membrane translocation of CLICs and mediate Cl^−^ efflux to regulate NLRP3 inflammasome assembly (Domingo-Fernández et al., 2017). Mitochondrial dysfunction and ROS-generating mitochondrial regulation are the other key upstream signals in NLRP3 activation (Zhou et al., 2011). Under normal physiological conditions, thioredoxin-interacting protein (TXNIP), an NLRP3-binding protein, interacts with thioredoxin (TRX) and blocks its activation. With the accumulation of ROS-generating mitochondria and ROS, TXNIP is released from the TRX complex and in turn binds NLRP3 to activate the NLRP3 inflammasome (Zhou et al., 2010). Lysosome rupture and Ca^2+^ release activate the Ca^2+^-CAMKⅡ-TAK1-JNK pathway, which regulates NLRP3 inflammasome activation through the oligomerization of ASC (Okada et al., 2014). However, the role of Ca^2+^ signaling in NLRP3 activation remains controversial. Various NLRP3 stimuli lead to the disassembly of the intracellular trans-Golgi network (TGN) into various dispersed structures, forming the dTGN. NLRP3 is recruited to the dTGN through negatively charged phosphatidylinositol-4-phosphate (PtdIns4P) on its ionic membrane. It serves as a scaffold for oligomerization of the adaptor protein ASC to aggregate into multiple puncta, thereby activating caspase-1 and downstream signals (Chen and Chen, 2018).

NLRP3 inflammasome activation contributes to the host’s defense against microbial infection. However, NLRP3 dysfunction leads to inflammatory disease. Thus, it is critical to precisely regulate NLRP3 inflammasome activation to provide adequate immune protection without damaging the host. NLRP3 inflammasome activation is precisely regulated by posttranslational modifications such as phosphorylation and ubiquitination (Kelley et al., 2019), as well as modification of NLRP3 by certain pathogen proteins such as S-nitrosylation and ADP-ribosylation (Mishra et al., 2013; Bose et al., 2014).

### AIM2

AIM2 is a major cytosolic dsDNA sensor consisting of the C-terminal HIN-200 domain and the N-terminal PYD (Jin et al., 2012). In the normal state, the HIN-200 domain and PYD interaction maintains the AIM2 receptor in an autoinhibited state in the absence of dsDNA (Jin et al., 2013). Cytoplasmic DNA derived from damaged host tissue, microbial or viral pathogens, can be sensed directly and combined with the HIN-200 domain of AIM2 to expose the AIM2 PYD and recruit the adaptor protein ASC, leading to activation of the ASC pyroptosome and caspase-1 (Fernandes-Alnemri et al., 2009; Hornung et al., 2009). The AIM2 inflammasome is essential for host defense against various pathogens, including bacteria, viruses, fungi, and parasites. However, several studies have shown that AIM2 is involved in the occurrence and development of aseptic inflammatory diseases such as atherosclerosis, chronic kidney disease, skin diseases, liver disease, and neuroinflammation (Sharma et al., 2019).

### NLRC4

NLRC4 belongs to the NLR family and consists of an N-terminal CARD, a C-terminal LRR domain, and an NACHT domain activated by bacterial flagellin and components of flagella-associated secretion systems (Poyet et al., 2001). However, NLRC4 recognizes these ligands indirectly, and NLRC4 inflammasome activation depends on NLR family apoptosis inhibitory protein (NAIP) (Kofoed and Vance, 2011). The binding of the ligand (microbial flagellin) to NAIP induces a conformational change leading to NLRC4 assembly, and exposure of its oligomerization interface is initiated to recruit and activate caspase-1 (Haloupek et al., 2019).

### NLRP1 and CARD8

NLRP1 was the first discovered member of the NLR family and is activated by a variety of stimuli, such as anthrax lethal toxin, *Shigella flexneri*, *Toxoplasma gondii*, and the small-molecule DPP8/9 inhibitor Val-boroPro (VbP) (Martinon et al., 2002; Ewald et al., 2014; Okondo et al., 2018; Sandstrom et al., 2019). Human NLRP1 consists of the PYD, NACHT domain, LRR domain, FIIND, and CARD, wherein the FIIND is a specific domain different from other NLR family proteins that can be autolytically cleaved and key for NLRP1 activity (D’Osualdo et al., 2011; Finger et al., 2012). CARD8 has an FIIND similar to NLRP1 but lacks the N-terminal domain in NLRP1. In general, the activation of NLRP1 and CARD8 is dependent on autolytic cleavage of the N-terminal peptide fragment, and activated NLRP1 recruits pro-caspase-1 to construct the NLRP1 inflammasome that cleaves pro-caspase-1 into active caspase-1 (Chavarría-Smith et al., 2016).

### Pyrin

Pyrin, a member of the TRIM protein family, can bind to the inflammasome adaptor protein ASC to form a caspase-1–activating inflammasome complex by sensing the pathogen, inactivating Rho GTPases, and decreasing the RhoA-dependent phosphorylation of pyrin, leading to pyroptosis (Xu et al., 2014; Park et al., 2016).

## Ocular Diseases, Inflammasomes, and Pyroptosis

**FIGURE 3:**
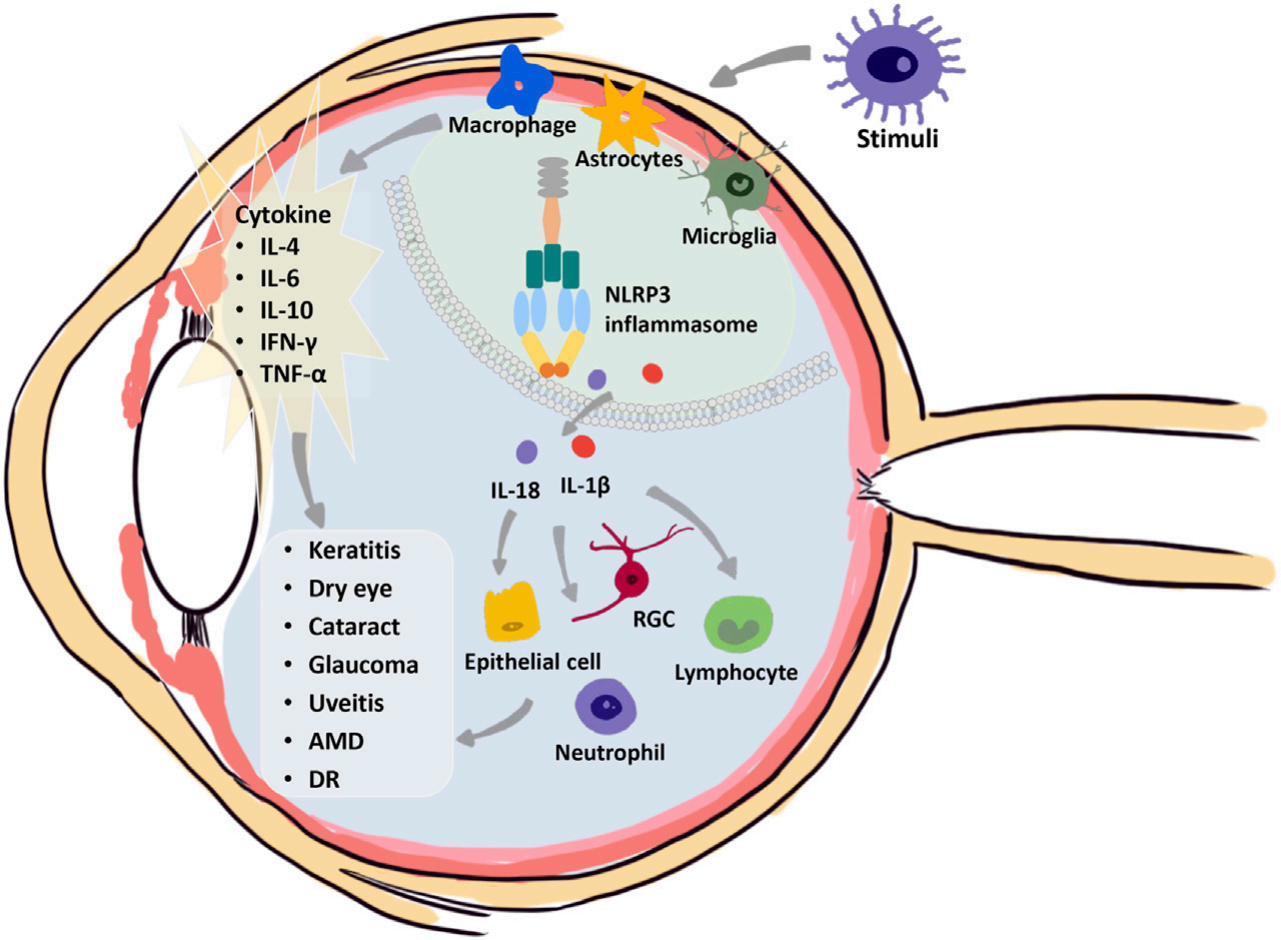
Crucial role of the NLRP3 inflammasome in the pathogenesis of eye diseases. The NLRP3 inflammasome mediates eye inflammation occurrence under various stimuli, such as bacterial and viral infections, desiccating stress, autoimmune factors, drusen, and complement proteins. Activation of immune cells results in a cascade of massive inflammatory cytokines, including IL-4, IL-6, IL-10, IFN-γ, and TNF-α. Meanwhile, NLRP3 inflammasome activation occurs in activated macrophages, astrocytes, and microglia, which drives the secretion of IL-1β and IL-18 to recruit inflammatory cells and induce the damage or cell death of different cells. Multiple damage factors induce various injuries in the different ocular tissues, leading to keratitis, dry eye, cataracts, glaucoma, uveitis, age-related macular degeneration, or diabetic retinopathy.

Ocular diseases involve in excessive or chronic inflammation. During the development of diseases, several cellular processes are activated, involving different cell deaths, including necroptosis, pyroptosis, ferroptosis, and autophagy, which can cause ocular tissue damage. DAMPs and PAMPs seem to be important connections between inflammation and cell death (Murakami et al., 2020). Although current studies advance the knowledge of the cell death mechanisms involved in ocular disorders, the diversity of consequences shows the complexity of various cell deaths that may be involved in disease pathogenesis. Further exploration of the value of different cell deaths in the pathogenesis of ocular disorders is warranted. It is now clear that inflammasomes have a central role in the pathogenesis of basically all types of chronic inflammation in metabolic, hereditary, systemic, and eye diseases (Figure 3). It acts as the processing unit to integrate the signals of pathogens, cell or tissue damage, and foreignness (Di Virgilio, 2013). The human eye has a well-developed immune surveillance system that can help fight against various pathogens, involving the contribution of NLRP1, NLRP3, NLRC4, NLRP12, and AIM2 inflammasomes (Gu et al., 2019; Pronin et al., 2019; Chen et al., 2020b; Lyon et al., 2020; Jassim et al., 2021; Yao et al., 2021). Unlike the protective effect of activated inflammasomes in combating pathogens in other tissue infections, such as infections of the lung with *Mycobacterium tuberculosis*, the activated inflammasomes in ocular diseases lead to increased severity of inflammation and more serious tissue destruction (Master et al., 2008; Akhtar-Schäfer et al., 2018). Aberrant inflammasome activation and IL-1β abundance upregulation have been shown to have no protective role in eye disease. Thus, in the eye, therapies that inhibit inflammatory body activation or IL-1β production may help to improve disease outcomes (Mesquida et al., 2019). Here, we emphasize the crucial role of pyroptosis and inflammatory inhibition in several ocular disorders, including keratitis, dry eyes, cataracts, glaucoma, uveitis, age-related macular degeneration, and diabetic retinopathy.

### Keratitis

Keratitis can easily cause corneal ulcers and even blindness. Various inflammasomes are assembled in response to bacterial, viral, fungal, and parasitic infections of the eye through DAMP/TLR signaling. A moderate inflammatory response contributes to resistance to infection by enabling leukocyte migration, but a severe and uncontrolled inflammatory response leads to corneal damage and ultimately corneal ulceration and perforation (Hazlett, 2004). Mouse macrophages infected with *Streptococcus pneumoniae* and *Pseudomonas aeruginosa* trigger NLRP3- and NLRC4-mediated inflammatory responses (Sutterwala et al., 2007; Karthikeyan et al., 2013). Pneumococcal hemolysin is a TLR4 ligand that mediates the activation of NLRP3 via TLR4 signaling (Malley et al., 2003). During inflammation, the assembly of inflammasomes and activated caspase-1 promote IL-1β release and attract inflammatory mediators such as cytokines and chemokines (Khan et al., 2007). In the course of pathogen elimination, cleaved GSDMD triggers cell pyroptosis, and corneal tissue may be damaged, resulting in partial or permanent blindness (Zhao et al., 2021).

The study indicated the involvement of non-canonical pyroptosis in *P. aeruginosa* keratitis, and the expression of caspase-4/5/11 and cleaved GSDMD in LPS-induced cell models, keratitis rat models, and *P. aeruginosa* keratitis patients was increased (Xu et al., 2021). Thus, targeting the activation of caspase-4/5/11 could inhibit the development of *P. aeruginosa* keratitis and suppress the release of proinflammatory cytokines. The protein GSDMD, as a universal substrate for inflammatory caspases, is an important performer of pyroptosis. Pretreatment with GSDMD siRNA attenuated the corneal inflammatory response significantly in mouse corneas infected with *Aspergillus fumigatus*, accompanied by decreased IL-1β secretion and attenuated recruitment of neutrophils and macrophages (Zhao et al., 2021). Inhibitors of IFNR, JAK/STAT, and caspase-1 can inhibit the expression of GSDMD, which may emerge as a potential therapeutic target (Zhao et al., 2021). In fungal keratitis, pretreatment with thymic stromal lymphopoietin (TSLP), a kind of inflammatory factor similar to IL-7 that is mainly produced by epithelial cells, promoted the expression of NLRP3, ASC, caspase-1, GSDMD, IL-1β, and IL-18 in THP-1 macrophages, which was abolished by NLRP3 knockdown. TSLP induces caspase-1–dependent pyroptosis through activation of the NLRP3 inflammasome, suggesting that it may be a potential target for fungal keratitis (Ji et al., 2021).

### Dry Eye

Dry eye is a common ocular surface disease characterized by a loss of homeostasis of the tear film, causing eye irritation and even visual disturbance (Craig et al., 2017). Damage to the ocular surface and hyperosmolarity-triggered inflammation play important roles in dry eye pathogenesis, which impacts the ocular surface epithelium and resident immune cells (Pflugfelder and de Paiva, 2017). Multiple activated inflammatory pathways trigger the secretion of innate inflammatory molecules, which provoke goblet cell loss, reduce mucus secretion, and trigger apoptosis of epithelial cells, initiating a vicious self-perpetuation cycle to destroy the tear film (Wei and Asbell, 2014). The NLRP3 inflammasome interacts with the ASC protein to trigger caspase-1 activation and IL-1β and IL-18 maturity; in addition, activated caspase-1 induces GSDMD-driven cell pyroptosis (Swanson et al., 2019). GSDMD-driven pyroptosis in desiccating stress–induced dry eye mice has been demonstrated. Desiccating stress–induced TLR4 activation promotes NLRP12, and NLRC4 inflammasome–mediated GSDMD-dependent pyroptosis is responsible for processing bioactive IL-33, which exacerbates inflammation in dry eye via caspase-8 signaling (Chen et al., 2020b). Inhibition of NLRP12 or NLRC4, deletion of GSDMD, and neutralization of mature IL-33 significantly attenuated desiccating stress–induced corneal epithelial damage, suggesting that these components are potential therapeutic targets for dry eye disease (Chen et al., 2020b). A recent study examined the increased expression of the pyroptosis executor GSDMD N-terminal domain in tears from dry eye patients and demonstrated direct evidence of the involvement of pyroptosis in dry eye patients. Calcitriol can effectively alleviate hyperosmotic stress–induced corneal epithelial cell damage by inhibiting the NLRP3–ASC–caspase-1–GSDMD pyroptosis signaling pathway (Zhang et al., 2021).

### Cataract

Cataracts, the loss of lens transparency, are the major cause of blindness in the world, accounting for half of all blindness (Flaxman et al., 2017). Although it can be treated by surgically removing the opaque lens and implanting the artificial lens, it is essential to develop new therapeutic targets to prevent cataract progression. Several studies have demonstrated that pyroptosis might have a role in cataract formation and that the levels of pyroptosis markers, such as NLRP3, pro-caspase-1, active caspase-1, GSDMD-N, IL-1β, and IL-18, are significantly increased in the capsule tissues or cells of cataract patients (Sun et al., 2020). Furthermore, active caspase-1 expression was increased in lens epithelium cells treated with H_2_O_2_, which was downregulated by using caspase-1 inhibitors, followed by pyroptosis inhibition. H_2_O_2_-induced oxidative stress could activate NF-κB signaling and increase NLRP3 expression significantly in human lens epithelial cells via caspase-1 activation and maturation of IL-1β, which contributes to pyroptosis and the development of cataracts (Jin et al., 2018). Human lens epithelial cells (LECs) irradiated with UVB induced pyroptosis, in which the expression of NLRP3, active caspase-1, pro-caspase-1, and GSDMD-N was increased significantly (Sun et al., 2020). Moreover, a high level of CRTAC1 plays an important role in pyroptosis in cataracts, while the downregulation of CRTAC1 significantly attenuates UVB-induced pyroptosis (Sun et al., 2020). In addition to the canonical inflammasome pathway, non-canonical inflammasomes are involved in caspase-11–triggered and GSDMD-triggered cataracts in a pyroptosis-dependent manner in short-wavelength blue light–exposed rat lens cells (Wang X. et al., 2021). A recent study demonstrated that the expression of the pyroptosis markers caspase-1, caspase-11, and GSDMD in rat LECs was increased after short-wavelength blue light exposure (Wang et al., 2020).

### Glaucoma

Glaucoma is a neurodegenerative disease characterized by the loss of retinal ganglion cells (RGCs), thinning of the retinal nerve fiber layer, and cupping of the optic disc (Weinreb et al., 2014). Several recent studies have supported the crucial roles of inflammation in the pathogenesis of acute glaucoma (Baudouin et al., 2021). Inflammation activation primarily occurs in the optic nerve head (ONH) and retina, in which activated astrocytes, activated microglia, and proinflammatory cytokines (IL-1β) are detected (Sun et al., 2013; Bordone et al., 2017; Williams et al., 2017). Activated inflammation interacts with oxidative stress, such as reactive oxygen species (ROS) and nitric oxide (NO), and promotes an increase in the amount of each other, creating a chronically activated state of inflammation (Adornetto et al., 2019; Fernández-Albarral et al., 2021). The increased inflammatory cytokines IL-1β, IL-4, IL-6, IL-10, and IFN-γ, in turn, led to a reduction in RGCs in the glaucoma model, which might be associated with microglial activation (Bosco et al., 2015).

Dysregulation of the NLRP3 inflammasome has been associated with various neurodegenerative diseases. One study has shown that the NLRP3 inflammasome, caspase-1, and caspase-8 are increased in human glaucoma compared to normal eyes (Yang et al., 2011). Clinical studies have observed an upregulation level of IL-1β mRNA and protein expression in the blood of glaucoma patients, suggesting activation of the NLRP3 inflammasome in glaucoma (Markiewicz et al., 2015). In an inducible mouse model of acute glaucoma, microglia-induced pyroptosis-mediated RGC death was associated with glaucomatous vision loss (Chen et al., 2020a). Caspase-8 plays a critical role in IOP-induced cell death. TLR4 signaling, mediated by caspase-8, is essential to activate NLRP3 inflammasomes and process pro-IL-1β (Chi et al., 2014). Targeted inhibition of TLR4 and caspase-8 signaling significantly blocked the production of IL-1β and attenuated RGC death and retinal ischemic damage (Chi et al., 2014). NLRP12 collaborates with NLRP3 and NLRC4, downstream of the caspase-8–HIF-1α axis, to trigger pyroptosis by GSDMD cleavage and IL-1β maturation through caspase-1 activation (Chen et al., 2020a). High-mobility group box 1 (HMGB1) protein is released, mediated by rapid IOP elevation, triggering caspase-1–dependent NLRP3 inflammasome activation and IL-1β production via caspase-8 (Chi et al., 2015).

### Uveitis

Uveitis is a complex ocular inflammatory disease usually caused by underlying autoimmune diseases (non-infectious/autoimmune uveitis) or bacterial or viral infections (infectious uveitis) (Shome et al., 2021). Current therapies concentrate on topical corticosteroids to achieve rapid remission of initial inflammation, followed by treatment with immunosuppressants to control long-term chronic inflammation (Lee K. et al., 2014). The inflammatory pathway, especially the role of the inflammasome, is highlighted as a responsibility for the development of uveitis. Most cells express one or more NLRs and contain the machinery to form inflammasomes, including NLRP1–4 (Lim et al., 2020). Activated inflammasomes, especially NLRP3, as well as the proinflammatory cytokines IL-1β and IL-18, have been implicated in uveitis. The imbalance of the adaptive immune system, especially macrophages, myeloid cells, and activated T helper cells, is credited for chronic uveitis. Th1, Th2, Th17, and T regulatory cells (Tregs) are diverse subsets of naïve T cells after stimulation by cytokines and transcription, which can lead to cytokine release in turn and upregulate lymphocyte infiltration to exacerbate inflammation (Lee RW. et al., 2014). Recent studies have indicated that the inflammasome might play an important role in the differentiation, expansion, and survival of Th17 cells (Mills et al., 2013). IL-1β produced by activated inflammation with IL-23 induces the secretion of IL-17 from Th17 cells (Sutton et al., 2006). Treatment of mice with IL-1β along with retinoid-binding protein injection enhanced experimental autoimmune uveitis development, whereas treatment with an anti-IL-1β antibody attenuated the inflammatory response (Zhao et al., 2014). IL-1 receptor–deficient mice showed better tolerance to the experimental autoimmune uveitis animal model, further confirming the importance of IL-1β in autoimmune uveitis (Wan et al., 2016). As a novel target for autoimmune uveitis, IL-6 impacts the differentiation and stimulation of Th17 cells with TGF-β and IL-23, while considering that IL-1β is necessary for the production and regulation of IL-6, the inflammasome might be highly involved in the development of uveitis (Rose-John, 2012). These studies reveal major molecular mechanisms of inflammasome assembly during inflammatory processes. However, there is no clinical evidence for the role of inflammasomes and pyroptosis in human uveitis. Most of the evidence for inflammasome assembly and pyroptosis comes from cellular and animal models. Therefore, further exploration of the role of the inflammasome and pyroptosis in uveitis is required.

### Age-Related Macular Degeneration

Age-related macular degeneration (AMD) is a degenerative disease leading to progressive photoreceptor loss and retinal pigment epithelium (RPE) damage. Inflammation has a role in AMD pathogenesis (Ambati et al., 2013). The NLRP3 inflammasome can be activated by drusen, RPE, complement protein, nucleic acid, oxidative stress, and its products beyond homeostasis-maintaining parainflammation (Tseng et al., 2013). Drusen, as a hallmark of AMD progression, has a rich proteinaceous and potentially damaging composition that triggers potential interactions with the NLRP3 inflammasome, including lipids, lipoproteins, RPE-derived cellular debris, e.g., organelles, melanin granules, lipofuscin, amyloid-β (A*β*), apolipoprotein E, and oxidation byproducts, as well as numerous inflammation-related factors, such as complement components, immunoglobulins, HLA molecules, and acute phase proteins (Hageman et al., 1999; Hageman and Mullins, 1999; Crabb et al., 2002; Sakaguchi et al., 2002; Johnson et al., 2011).

The chronic, sustained pathological simulated stage of the complement system could generate activated complement factors, promoting the formation of a terminal membrane attack complex to intensify the AMD pathological course (Khandhadia et al., 2012). Drusen extracts isolated from AMD donor eye tissues are able to activate the NLRP3 inflammasome, wherein complement factor 1q, as an activated signal, indirectly induces caspase-1 cleavage and increases IL-1β secretion in human monocytic THP-1 cells (Doyle et al., 2012).

Amyloid-β, a component of drusen in AMD eyes, contributes to inflammasome activation and AMD pathological progression. A*β* was discovered to be produced by cleaving the intramembranous proteolysis of amyloid precursor protein as a pathologically neurotoxic peptide, showing specific deposition within drusen in AMD eyes (Haass and Selkoe, 2007). In the RPE cell culture model, A*β* promotes RPE gene expression changes in pathways associated with inflammation and the immune response (Kurji et al., 2010). It also stimulates upregulation of the IL-1β, IL-6, IL-18, caspase-1, and NLRP3 genes in the retina using intravitreal A*β* injection in rat models, which demonstrates NLRP3 inflammasome activation (Liu et al., 2013). Mice with caspase-1 knockout showed increased photoreceptor survival and better protected retinal function with an attenuated inflammatory response (Hanus et al., 2015). With prolonged inflammation of the retina, RPE cells became enlarged or swollen and exhibited a significant increase in proteolysis of full-length GSDMD in RPE choroidal tissue, and the expression of GSDMD-N, caspase-1, IL-1β, and IL-18 was also significantly increased in intravitreal A*β*-induced AMD models (Gao et al., 2018; Sun et al., 2018). Earlier studies have reported that the cytolytic effect of pyroptosis is mediated by the oligomerization of the N-terminal fragment (N-GSDMD) of GSDMD on the cellular membrane, leading to the formation of cellular rupture pores (Liu X. et al., 2016; Sborgi et al., 2016). Hence, this confirms the activation of the pyroptotic pathway in the Aβ-injected models. These studies illustrated the existence of A*β*-induced AMD models with secretion of mature proinflammatory cytokines, including inflammatory factors (IL-18 and IL-1β), and evidence supporting GSDMD-mediated activation of the pyroptosis pathway in RPE cells, suggesting the importance of pyroptosis in AMD pathogenesis, while inhibiting the target of the pyroptosis pathway may benefit the prognosis of AMD.

Another trigger of inflammasomes is oxidation byproducts. In a normal physiological state, RPE cells phagocytose the deciduous outer segments of photoreceptors. However, the ability of RPE cells to recycle “waste” from photoreceptors significantly decreased with age, resulting in the accumulation of the lipid peroxidation byproduct lipofuscin in the RPE (Schütt et al., 2002). Deposition of lipofuscin in RPE causes lysosomal damage and directly triggers the activation of the NLRP3 inflammasome, as well as other lipid peroxidation end products, such as 4-hydroxynonenal and carboxyethylpyrrole (Doyle et al., 2012; Kauppinen et al., 2012; Tseng et al., 2013).

### Diabetic Retinopathy

Chronic inflammation plays a crucial role in diabetic retinopathy (DR) pathology (Tang and Kern, 2011). NLR-mediated inflammatory pathways function via NF-κB signaling, which is a transcription factor that regulates proliferation, angiogenesis, apoptosis, and the immune response (Xie et al., 2010; Motta et al., 2015). Moreover, NF-κB is a known regulator of IL-1β expression and can modulate the activation of the NLRP3 inflammasome (Bauernfeind et al., 2009). High expression of NLRP3, caspase-1, and IL-1β is observed in retinal proliferative membranes and vitreous samples obtained from DR patients with vitreoretinal surgery (Loukovaara et al., 2017; Zhang et al., 2017). STZ-induced diabetic mice with DR features exhibit higher levels of NLRP1 and NLRP3. Consistently, NLRP1^−/−^ DR mice attenuated the expression of ASC and caspase-1 and further markedly decreased retinal NF-κB, IL-1β, and IL-18 levels compared to WT/DR mice (Li et al., 2018). In spontaneously hypertensive rats fed a high-fat diet to form proinflammatory state models, the interaction of TXNIP–NLRP3 and the expression of cleaved caspase-1 and cleaved IL-1β were markedly increased, indicating that TXNIP is required for NLRP3 inflammasome activation and IL-1β release (Mohamed et al., 2014). High glucose–induced TXNIP activation in retinal endothelial and Müller glial cells was also shown to promote oxidative stress (Devi et al., 2013). The TXNIP^−/−^ DR mouse model showed attenuated NLRP3 activation and retinal inflammation (Perrone et al., 2010). In short, NLRP3 plays a significant role in perpetuating inflammation in DR.

Retinal microglial and Müller cell activation contributes a major role in the onset of inflammatory processes in the early stages of DR. *In vitro*, primary or immortalized Müller cells treated with high glucose all have elevated NLRP3 protein expression (Li et al., 2019). Mouse RGCs treated with high glucose also upregulated NLRP1 and NLRP3 expressions, which were attenuated with the TLR4 inhibitor TAK-242 (Hu et al., 2017; Li et al., 2018). Mitochondria play a significant role in DR, in which damaged mitochondrial DNA (mtDNA) and impaired transcription of mtDNA genes are released into the cytosol, directly activating the NLRP3 inflammasome (Shimada et al., 2012; Kowluru, 2019). Augmented mitophagy/autophagy could lead to mitochondrial dysfunction and excessive ROS production, which is associated extensively with NLRP3 activation (Zhang W. et al., 2019).

Recent studies have demonstrated that a chronic hyperglycemic state induces GSDMD-mediated pyroptosis, which plays an important role in DR development. A high glucose state can stimulate the NLRP3–caspase-1–GSDMD signaling axis, which further promotes plasma membrane pore generation and inflammatory factor (IL-1β and IL-8) secretion in human retinal progenitor cells (HRPs) (Gan et al., 2020b). In addition, activated NLRP3–caspase-1–GSDMD–dependent pyroptosis induces retinal pericyte loss in a concentration-dependent manner in high-glucose surroundings (Gan et al., 2020b). GSDMD gene silencing markedly prevented HRP pyroptosis by inhibiting the NLRP3/caspase-1/GSDMD signaling axis. Another study showed that inhibition of caspase-1 can decrease the GSDMD protein and attenuate the release of IL-1β, IL-18, and LDH in a serum albumin–induced DR environment, illustrating the appearance of caspase-1–dependent pyroptosis in HRPs (Yu et al., 2021). P2X7R, as an oxidative stress and metabolic sensor, contributes to NLRP3 inflammasome activation and is distributed in the retinal microvascular epithelium, neural cells, and macrophages. P2X7R indirectly affected advanced glycation end product–induced retinal microvascular endothelial cells via the NLRP3 inflammasome. Meanwhile, the inhibition of P2X7R significantly reduced the expression and activation of the NLRP3 inflammasome, indicating that the initiation of NLRP3 inflammasome activation is dependent on the activation of P2X7R mediated by AGE–BSA–induced diabetic retinopathy (Yang K. et al., 2020).

## The Pyroptosis Inhibitors in Ocular Disorders

Inflammasomes involved in the processes of ocular pathogenesis play crucial roles in various eye diseases. Although the exact role of the inflammasome and its interacting proteins in human eye disease has not been fully elucidated, small-molecule inhibitors targeting the inflammasome and the pyroptosis process in human eye disease could help to design effective therapies assisting clinical practice. Excitingly, several pyroptosis inhibitors have been reported, including NLRP3 inhibitors, caspase-1 inhibitors, GSDMD inhibitors, and indirect inhibitors targeting inflammasome components or related signaling events. However, some pyroptosis inhibitors have yet to be tested in human or animal models of eye disease, and even some inhibitors tested in eye disease have a potential risk due to their incompletely elucidated precise target of inhibitory mechanisms. Furthermore, in the process of eye disease development, pyroptosis may have different weight values in different stages. For example, at the early stage of retinal neovascularization formation, the expression of NLRP3 and caspase-1 begins to increase and is significantly exacerbated later, while it shows a relatively low expression level at the stage of neovascular regression (Sui et al., 2020). Similarly, the expression of activated caspase-1 and activated IL-1β was also inconsistent at the time point. These factors may be associated with various factors, including the functional efficiency of activated caspase-1, pro-IL-1β expression, the degradation degree of activated IL-1β, and other complex regulatory mechanisms in biological individuals. These factors might determine the time point of treatment to intervene in disease development. Inhibition of NLRP3, caspase-1, or GSDMD specifically to reverse the activation pattern of pro-IL-1β or pro-caspase-1 might have a protective effect on eye diseases. Here, we summarize the pyroptosis inhibitors that have been applied to animal models of eye disease (Table 1). MCC950, as the most potent and specific NLRP3 inhibitor, specifically inhibits canonical and non-canonical NLRP3 inflammasome activation in both human and mouse macrophages *in vitro* without impacting the other inflammasomes and strongly attenuates the release of inflammatory factors both *in vivo* and *in vitro* (Coll et al., 2019). INF39 inhibits NLRP3 inflammasome activation by inhibiting NLRP3 ATPase enzyme activity but with poor specificity (Cocco et al., 2017). The marketed drug “tranilast” directly binds to NLRP3 to inhibit NLRP3 polymerization and activation (Huang et al., 2018). β-Carotene was discovered to bind to the PYD of NLRP3 to block the direct interaction of NLRP3 and ASC to inhibit inflammasome complex formation (Yang G. et al., 2020). The caspase-1 inhibitor VX-765 could inhibit the maturation of the two cytokines IL-1β and IL-18 and reduce the secretion of inflammatory cytokines and chemokines (Wannamaker et al., 2007). The GSDMD inhibitors necrosulfonamide and disulfiram covalently bind to Cys^191^ on GSDMD to block pore formation and inflammatory factor release and inhibit pyroptosis progression (Rathkey et al., 2018). Other indirect inhibitors of the inflammasome, including related ion channel inhibitors, TLR4 inhibitors, P2X2R inhibitors, HSP90 inhibitors, and some small-molecule inhibitors impacting related signaling events, have been tested in eye disease models *in vivo* and *in vitro*. With thorough studies of the inflammasome and pyroptosis in various eye diseases and an increased number of individuals affected by inflammatory disease, direct and specific pyroptosis inhibitors will boost future clinical translation, epitomizing the use of precision medicine in inflammasome-related diseases.

**TABLE 1:** Pyroptosis inhibitors in eye disease.

Inhibitor	Structure	Mechanism	Animal models of eye disease	References
**NLRP3 Inhibitors**				
MCC950	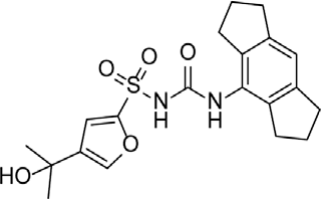	Specifically, it inhibits canonical and non-canonical NLRP3 inflammasome activation *in vitro*. Its central sulfonylurea group interacts with the Walker A motif of the NLRP3 nucleotide-binding domain	Ocular hypertension	Zhang et al. (2019b), Sui et al. (2020), Wooff et al. (2020)
			Oxygen-induced ischemic retinopathy	
			Photo-oxidative damage-induced retinal degeneration	
INF39	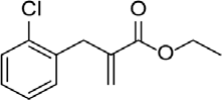	NACHT ATPase inhibitor. Reduces NLRP3, IL-1β, and caspase-1 expressions and attenuates the pyroptosis level	-	Huang et al. (2020)
Tranilase	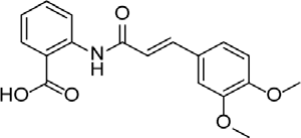	Binds NACHT domain and inhibits the NLRP3–NLRP3 interaction and the expression of cytokines and chemokines	-	Liu et al. (2016b), Yumnamcha et al. (2019)
β-Carotene	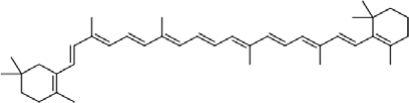	Protects the eye from oxidative stress, apoptosis, mitochondrial dysfunction, and inflammation	-	Camelo et al. (2020), Johra et al. (2020)
**Caspase-1 Inhibitor**				
VX-765	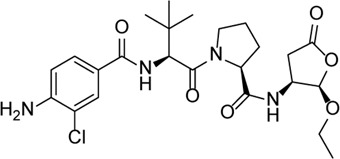	Decreases caspase-1 and inhibits the mature IL-1β and IL-18 and caspase-1–mediated pyroptosis	-	Lin et al. (2018), Li et al. (2021)
**GSDMD Inhibitors**				
Necrosulfonamide	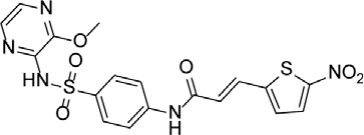	Inhibits the expression of NLRP3 and GSDMD and reverses the effects of high glucose on ARPE-19 cell proliferation	-	Xi et al. (2020)
Disulfiram	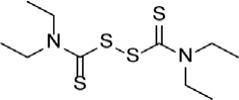	Blocks GSDMD pore formation, reduces the proportion of pyroptotic cells, and prevents cells against hyperosmotic stress–induced cytotoxicity	Diabetic retinopathy (OLETF rats)	Ito et al. (2010), Kanai et al. (2010), Kanai et al. (2012), Deguchi et al. (2020), Zhang et al. (2021)
			Ocular hypertension induced by rapid infusion of 5% glucose solution	
			Endotoxin-induced uveitis	
**Other Indirect Inhibitors of the Inflammasome**				
Glyburide	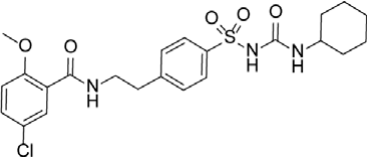	Inhibition of NLRP3 inflammasome activation and release of IL-1β by changing ion channel activity	Alu RNA–mediated geographic atrophy	Kerur et al. (2013), Gan et al. (2020a)
Ticagrelor	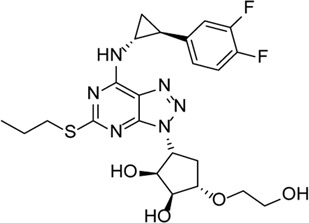	Induces degradation of Cl^−^ channel to block chloride outflow and inhibits the interaction of NLRP3 with ASC to inhibit activation of the NLRP3 inflammasome	ABCA4^−/−^ mouse model of retinal degeneration	Lu et al. (2018), Lu et al. (2019), Huang et al. (2021)
TAK-242	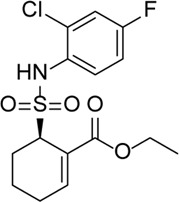	TLR4 inhibitor, which decreases the expression levels of TLR4 downstream signaling molecules (MyD88, NF-κB, TRAF6, NLRP3) and inflammatory factors (IL-1β and IL-18)	-	Hu et al. (2017)
N-Acetylserotonin	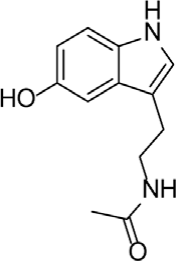	TLR4 inhibitor, which alleviates the expression of IL-1β in retinal ischemia–reperfusion rats via the TLR4/NF-κB/NLRP3 pathway	-	Liu et al. (2021)
A740003	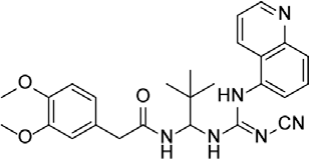	P2X7R inhibitor, which inhibits the activation of NLRP3 inflammasome and phosphorylation of IKBα	Oxidized low-density lipoprotein model (retinal inflammation and neovascularization)	Yang et al. (2021)
A438079	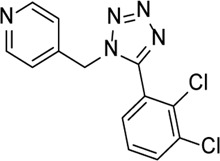	P2X7R inhibitor, which inhibits P2X7R–NLRP3 pathway reduced NLRP3 inflammasome expression	Ocular hypertension	Sakamoto et al. (2015), Zhang et al. (2019b)
			N-Methyl-d-aspartic acid–induced retinal injury	
TAS-116	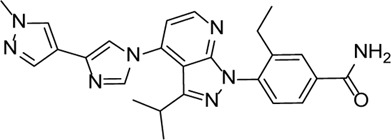	HSP90 inhibitor, which prevents the activation of caspase-1, subsequently reducing the release of mature IL-1β	-	Ranta-Aho et al. (2021)
Xanthone	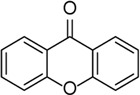	Inhibits cross-link ASC oligomerization, endogenous NLRP3 oligomerization, the cleavage of GSDMD, and the release of IL-1β	LPS-induced keratitis	Cui et al. (2021)
Verapamil	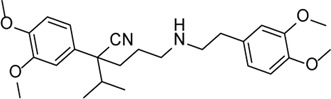	Inhibition of thioredoxin-interacting protein and inflammasome assembly	STZ-induced diabetic retinopathy	Eissa et al. (2021)
Calcitriol	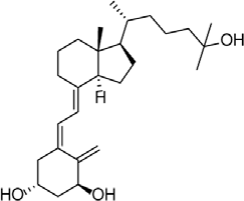	Alleviates hyperosmotic stress–induced corneal epithelial cell damage through inhibiting the NLRP3–ASC–caspase-1–GSDMD pyroptosis pathway	Corneal wound in STZ-induced diabetic mice	Wang et al. (2021b), Zhang et al. (2021)
Butyrate	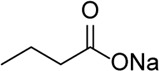	Decreased NLRP3, caspase-1, and IL-1β mRNA transcripts and NLRP3 protein expression	Corneal alkali burn	Bian et al. (2017)
Epigallocatechin-3-gallate	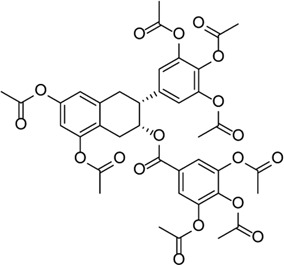	Inhibits the ROS/TXNIP/NLRP3 inflammasome axis to reduce ROS accumulation, NLRP3 inflammasome activation, Müller cell proliferation, and production of the pro-angiogenic factors	STZ-induced diabetic retinopathy	Du et al. (2020)

OLETF rats, Otsuka Long Evans Tokushima Fatty rats; LPS, lipopolysaccharide; STZ, streptozotocin.

## Conclusion

In this review, we summarize the mechanisms and functions of pyroptosis and inflammasomes in various ocular disorders. Clearly, our current knowledge of pyroptosis occurring in eye disease is just the tip of the iceberg. Many questions remain to be answered. Inflammasomes play a crucial role in the pathogenesis of eye disease, and pyroptosis could aggravate inflammation and cellular lysis death. However, most of the evidence available on inflammasome assembly and pyroptosis occurrence in eye diseases derives from *in vitro* and experimental models in animals, and inhibitors targeting inflammasomes and pyroptosis are currently being tested at the level of preclinical trials. It is essential to understand the role of inflammasomes and pyroptosis in human eye diseases and whether the data from experimental models are translatable to suit humans. In sum, we highlighted the increasing evidence about the role of pyroptosis in various eye diseases. In the future, the application of pyroptosis inhibitors may help us design new classes of targeting therapeutic agents to combat eye diseases.
